# Prevalence of depression and associated factors among people living with HIV/AIDS in public hospitals of Southeast Ethiopia

**DOI:** 10.1186/s12888-022-04205-6

**Published:** 2022-08-19

**Authors:** Fikreab Desta, Alelign Tasew, Yohannes Tekalegn, Demisu Zenbaba, Biniyam Sahiledengle, Tesfaye Assefa, Wogene Negash, Anwar Tahir, Tadele Regasa, Ayele Mamo, Zinash Teferu, Damtew Solomon, Habtamu Gezahegn, Kebebe Bekele, Zegeye Regassa, Daniel Atlaw

**Affiliations:** 1 Public Health Department, Goba Referral Hospital, Madda Walabu University, Bale Goba, Ethiopia; 2 Nursing Department, Madda Walabu University, Goba Referral Hospital, Bale Goba, Ethiopia; 3 Biomedical Unit, Madda Walabu University, Goba Referral Hospital, Bale Goba, Ethiopia; 4 Pharmacy Department, Madda Walabu University, Goba Referral Hospital, Bale Goba, Ethiopia; 5 Surgery Department, Madda Walabu University, Goba Referral Hospital, Bale Goba, Ethiopia

**Keywords:** Depression, ART, HIV/AIDS, Bale Zone, Ethiopia

## Abstract

**Background:**

Depression is the most frequent mental health condition among human immune deficiency virus or acquired immune deficiency syndrome (HIV/AIDS) patients. It has been related to negative health outcomes. This could lead to hospitalization and an increase in medical expenses. This study aimed to assess the prevalence of depression and associated factors among HIV/AIDS patients in public hospitals Bale Zone, Southeast Ethiopia.

**Methods:**

A hospital-based cross-sectional study design was randomly employed among 554 study participants. A systematic random sampling technique was used to select the study subjects. A structured Patients Health Questionnaires- 9 was used to measure the depression status of HIV/AIDS patients. Data were collected using a pretested interviewer administered structured questionnaire as well as review of patients medical charts or records. Descriptive statistics were computed. Multivariable logistic regression analyses were conducted identify factors associated with the prevalence of depression. Adjusted odds ratio (AOR), along with a 95% confidence interval (CI), was used to estimate the strength of the association. A *p*-value of < 0.05 was considered statistically significant.

**Results:**

The prevalence of depression among the study participants was found to be 44.9% (95% CI: 40.79%, 49.1%). Perceived HIV related stigma is the single most dominant predictor of depression [(AOR = 8.2, 95% CI: (4.96, 13.68)], low income level [(AOR = 3.1, 95% CI: (1.59, 6.22)] Experiencing any form of a side effect of highly active anti-retroviral therapy (HAART) [(AOR = 1.5, 95% CI: (1.04, 2.56)], having normal BMI [(AOR = 0.49, 95% CI: (0.29, 0.8)] being HIV patients at WHO clinical stage II [(AOR = 0.44, 95% CI: (0.22, 0.9)], were significantly associated with prevalence of depression.

**Conclusion:**

The study revealed that the prevalence of depression among people living with HIV in the study settings was high, almost two out of every five HIV patients were depressed. Low income level, side effect to HAART, and having HIV related stigma were more likely to suffer from depression.

## Background

Human immune deficiency virus or acquired immune deficiency syndrome (HIV/AIDS) continues to have serious health consequences around the world; with more than 36 million people currently living with HIV [1, 2]. Sub-Saharan Africa (SSA) accounts for 76% of total HIV infection in 2015 [3]. Ethiopia is one of SSA an estimated 669,236 people live with HIV in 2019 [4].

Individuals infected with HIV struggle with psychosocial influences, like poverty, stigma, depression, etc [5]. Depression is a common mental health problem among people with HIV/AIDS which is manifested by the sign and symptoms of poor appetite, sadness, sleep disturbance, poor concentration and feelings of exhaustion [6].

Depression is a major cause of disability; it contributes to almost 7.5% of all disabilities [7]. Currently, the burden of depression has been rising, and it will be the third leading cause of disease burden by 2030 [8–10]. Globally, over 300 million people, which is equivalent to 4.4% of the world’s population live with depression [11, 12]. Despite the fact that effective treatments for mental diseases exist, more than 75% of people in low- and middle-income countries (LMICs) do not receive them [13].

Depressive disorders affect 12% to 66% of HIV-positive people, with 50–60% going through undiagnosed [14]. Despite the inconsistencies, a meta-analysis found that co-morbid depression is nearly twice as common mong HIV-positive people (PLWH) [15]. Evidence has suggested that nearly 47% of the participants had depression problems [16]. Across LMICs prevalence of depression among adult HIV/AIDS patients ranged from approximately 13% to 78% [17]. A systematic review and meta-analysis conducted in PLWH in East Africa have demonstrated that the prevalence of depression among PLWH was 38% [18].

People living with HIV/AIDS suffer from depression as they adapt to life with a chronic condition, experience or anticipate stigma, or manage ongoing life stressors [19]. Although the development of antiretroviral therapy (ART) regimens has considerably enhanced the life expectancy of HIV/AIDS patients, they remain prone to mental diseases, particularly depression, due to the persistence of some psychosocial stresses [20].

There are a small number of studies conducted in different parts of Ethiopia that reveal an inconsistent and inconclusive prevalence of depression among PLWH/AIDS. Studies conducted between 2012 and 2016 years [21–25] indicated different level of prevalence of depression ranges from 14.6% [22] to 48.6% [23]. On the other hand, the recent studies [26–28] focused at urban and single Hospital. Even though, there are a few recently published studies [26–28] in Ethiopia on the prevalence of depression and the factors linked to depression in a PLWH/AIDS, to dates there is no published literature at the study area. Therefore, we aimed to assess the prevalence of depression and associated factors among HIV-infected people receiving ART from public health hospitals in the Bale Zone, Southeast, Ethiopia.

## Methods

### Study area and period

The study was conducted in Bale Zone public hospitals southeast Ethiopia. It is located about 403 km away from the capital of the country, Addis Ababa. The Bale zone has three hospitals, namely Goba Referral Hospital and two general hospitals (Dellomena and Robe General Hospital), that are currently giving ART service in the zone. There were about 3,308 adult HIV/AIDS patients who had registered for ART follow-up in public hospitals during the study period from February 1^st^ to April 30^th^, 2021.

### Study design

A hospital-based cross-sectional study design was applied to assess prevalence of depression and associated factors among people living with HIV/AIDS on ART following clinic.

### Source population

All adult aged ≥ 18 years HIV positive patients who had ART treatment follow up in public hospitals of Bale Zone.

### Study population

All randomly selected adult aged ≥ 18 years HIV positive patients who had treatment follow up during the study period in public hospitals of Bale Zone.

### Inclusion and exclusion criteria

#### Inclusion criteria

The study included patients who were receiving highly active antiretroviral therapy for at least six months.

#### Exclusion criteria

Patients who were unable to converse and had a serious general medical condition were excluded.

### Sample size determination

The sample size was determined by using the single population proportion formula using EPI Info version 7.2. using an assumption of 95% level of confidence, 4% marginal error and 48.6% proportion of depression among PLWHA [29], and after adding 10% non-responses which gave a total sample size of 559.

### Sampling procedure

**Fig. 1:**
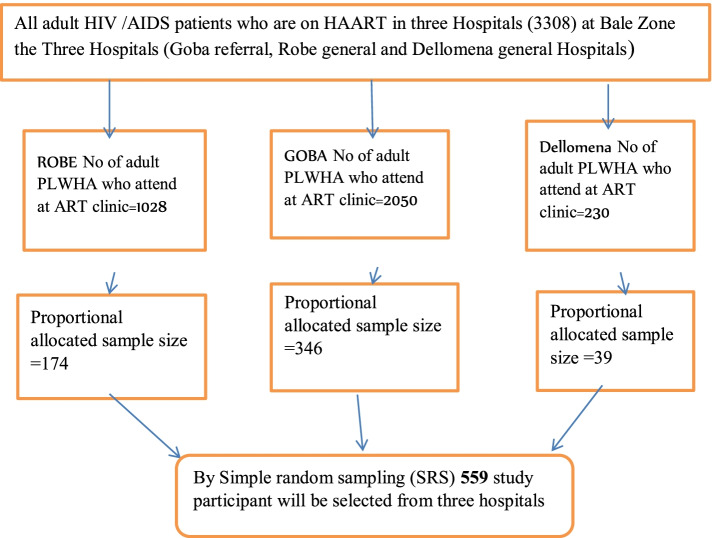
Schematic representation of sampling procedures of people living with HIV/AIDS on ART following clinic in public hospital, Bale Zone, Southeast, Ethiopia, 2021 (*N* = 559)

The study participants were chosen using a systematic random sampling technique. The sampling interval (k = 6) was calculated by dividing the patients on ART 3,308 people during data collection. The total sample size of 559 and the fourth patient was chosen at random (lottery method) from the first six ART patients, and then every six patients were included in the study. The sample size was allocated proportionally to each hospital after obtaining lists of potential participants from the ART register of each hospital (Fig. 1).

### Variables of the study

#### Dependent variable

Depression.

#### Independent variables

Socio-demographic and economic variables (age, sex, religion, residence, marital status, education level, occupation and monthly income).

Psychosocial variables (living condition, HIV related stigma, social support and lost job).

Clinical related factors (WHO HIV/AIDS stage, Current CD4 count, Medication adherence, drug regimen, current drug side effect, duration of HAART treatment, viral load).

### Operational definition

#### Depression

Depression was assessed by using Patient Health Questionnaire-9 (PHQ-9) that had total sum score of 27 from 9 items; those respondents who score 10 and above from total sum were considered as depressed while respondents who score below 10 was considered as non-depressed [30].

#### HIV related stigma

Perceived HIV related stigma was assessed by using HIV related Stigma scale that had a total sum score of 50 from 10 items; those respondents who score higher than the mean from total considered had perceived stigma while respondents who score lower than the mean considered as had no perceived stigma [31, 32].

#### Social support

Social support was assessed by using social support questionnaire (SSQ-6) that has a total sum score of 6 items; those respondents who score higher than the mean considered as good social support while respondents who score lower than the mean considered as to have poor social support [33].

#### Adherence status

Adherence was assessed by using a Morisky Medication Adherence Scale questions with a total sum score of 4 from 4 items; those who score 0 were considered as non-adherent while those who score one and above considered as adherent [34].

#### Side effects of ART

Respondents reported their side effects, including appetite changes, nausea and vomiting, difficulty sleeping, rash, numbness in the limbs, pain in the limbs, fatigue, body shape changes, hair loss and vision changes in their medical records.

### Data collection tool and procedure

A structured interviewer-administered questionnaire format with socio-demographic and economic characteristics (age, sex, residence, ethnicity, religion, marital status, educational level, monthly income, and occupation), psychosocial factors (HIV related stigma, social support, living condition, and lost jobs), and HIV/AIDS related factors (CD4 count, WHO HIV/AIDS stages, medication adherence, ART regimen, and duration on ART) was used to measure the association with depression among HIV/AIDS patients. Basic clinical information such as the current CD4 counts, the WHO clinical staging of HIV/AIDS, HAART regimen, and duration on ART was obtained from the respondents’ medical records. Side effect of drug were assessed based on respondents reporte to adverse reaction of drug, including appetite changes, nausea and vomiting, difficulty sleeping, rash, numbness in the limbs, pain in the limbs, fatigue, body shape changes, hair loss and vision changes in their medical records. Depression was assessed using the Patients' Health Questionnaires-9 (PHQ-9). It had an internal consistency (Cronbach’s alpha) of 0.85 [35]. Social support was assessed by (SSQ-6). It included a 6-item scale that assessed the availability of social support and its reliability coefficient for these items was 0.86 [36]. The HIV-related perceived stigma was also ascertained using the perceived stigma scale and its internal inconsistence of these items is 0.92 [37]. The questionnaire was prepared in English language originally and translated to Amharic and local language (Afaan Oromo) by language experts. The Amharic version was translated back to English to verify the consistency. Both Amharic and Afaan Oromo language questionnaire were used to collect data.

Data was collected using an interviewer administered questionnaire, and by extracting pertinent information from patient medical records, by four Bachelor Science (BSc) nurse in private rooms nearby the ART units. Prior to their participation in data collection, they were instructed on the purpose of the study, the ethical principles, how to fill out the questionnaire, and data management. The collection process was being supervised by principal investigators.

### Data quality control

Questionnaire was pretested. One-day intensive training was given on the objective of the study, confidentiality of information and interviewing technique. Every day the questionnaires was reviewed and checked for completeness, consistency and relevance by investigator.

### Data processing and analysis

The data were entered into Epi Data version 3.1 and being exported to Statistical Package for Social Science (SPSS) version 25 for cleaning, coding, and analysis. Descriptive statistics like, frequency, percentages, mean, and standard deviation were computed and presented by using text and tables. Bivariate regression was undertaken to see association between dependent and independent variables. Variables having a *p*-value of < 0.25 in Bivariate analysis were included in multivariable logistic regression model. The Hosmer–Lemeshow test was used to determine the final model's goodness of fit and the variance inflation factor (VIF) was used to check for multi-collinearity among selected independent variables. In the multivariable logistic regression model, variables having a *P*-value of < 0.05 was considered statistically significant.

## Results

### Socio-demographic and economic characteristics of study participants

An overall response rate of 99.1% was achieved with a total of 554 study participants. The mean age (± SD) of the respondents was 38.0 (with SD of ± 9.86 years). Among the study participants, 277 (50%) were female, 345 (62.3%) were married, and 201 (36.3%) were between the ages of 29–39 years. Out of all respondents, 167 (30.1%) had were attended primary education, 126(22.7%) were housewives and 405(73.1%) were living in rural areas (Table 1).

**Table 1:** Socio demographic characteristics of respondents in public hospitals of Bale zone, Southeast Ethiopia, 2021 (*N* = 554)

Variables	Frequency (*n* = 554)	Percent(%)
**sex**		
Male	277	50
Female	277	50
**Age**		
18–29	120	21.7
30–39	189	34.1
40–49	172	31
> = 50	73	13.2
**Residance**		
Urban	405	73.1
Rural	149	26.9
**Ethnicity**		
Oromo	365	65.9
Amhara	165	29.8
Others	24	4.3
**Religion**		
Protestant	93	16.8
Orthodox	242	43.7
Muslims	199	35.9
Catholic	17	3.1
Others	3	0.5
**Level of education**		
unable to read and write	105	19
able to read and write	61	11
Primary school	167	30.1
Secondary or preparatory	160	28.9
College and University	61	12.1
**Marital status**		
Single	68	12.8
Married	345	62.3
Widowed	74	13.4
Divorced	67	12.1
Occupation		
Farmer	128	23.1
Housewife	126	22.7
Government employee	116	20.9
Daily laborer	115	20.8
Others	69	12.5
**Income level**		
< = 500	95	17.1
501–1500	137	24.7
1501–2500	120	21
2501–3500	81	14.6
> = 3501	121	21.8
**Family size**		
< 5	454	81.9
5–7	79	14.3
> = 8	21	3.8

### Psychosocial and clinical related characteristics of respondents

More than half 377 (68.1%) of study participants have HIV related stigma and 381 (68.8%) have poor social support from their families or other supportive bodies. Most of respondents 438 (79.1%) were at WHO clinical stag I. Good adherence to HAART was reported by more than two-thirds of respondents 447(80.7%) (Table 2).

**Table 2:** Psychosocial, clinical and medication adherence related characteristics of respondents public Hospitals Bale zone, Southeast, Ethiopia, 2021 (*n* = 554)

Variables	Frequency(*n* = 554)	Percent (%)
**Living condition**		
Alone	91	16.4
With my wife	276	49.8
With family	166	30
Others	21	3.8
**Lost job due to HIV**		
Yes	156	28.2
No	398	71.8
**Opportunistic disease**		
no	473	85.4
Toxoplasma	15	2.7
Fungus	21	3.8
TB	34	6.1
Others^a^	11	2
**BMI category**		
< 18.5	110	19.9
18.5–24.99	395	71.3
25–29.99	46	8.3
> = 30	3	0.5
**Source of infection**		
Blood contact	95	17.1
Unsafe sexual intercourse	212	38.3
I do not know	239	43.1
Others^b^	8	1.4
**Comorbidities**		
No	480	86.6
Diabetes mellitus	17	3.1
Hypertension	27	4.9
Others^c^	30	5.4
**Substance use**		
Khat	89	16.1
Alcohol	137	24.7
Cigarettes	41	7.4
I do not use any	287	51.8
**Family member with HIV**		
< = 2	501	90.4
3–5	53	9.6
**HIV related stigma**		
Yes	377	68.1
No	177	31.9
**Social support**		
Poor social support	381	68.8
Good social support	173	31.2
**WHO clinical stage**		
Stage I	438	79.1
Stage II	68	12.3
Stage III	45	8.1
Stage IV	3	0.5
**CD4 count current**		
< 200	60	10.8
200–349	133	24
350–499	178	32.1
> = 500	183	33
**Adherence to medication**		
Poor adherent	107	19.3
Good adherent	447	80.7
**Drug side effect**		
Yes	154	27.8
No	400	72.2
**Drug regimen**		
First line	447	80.7
Second line	107	19.3
**Duration on HAART (in month)**		
< 12	16	2.9
13–24	28	5.1
> = 25	510	92

^a^pnemocicty crania pneumonia

^b^using sharp material commonly

^c^tuberculosis

### Prevalence of depression among PLWH

The prevalence of depression among PLWH/AIDS was assessed by PHQ-9. Based on the cut of point ≥ 10, the prevalence of depressive symptoms was found to be 249 (44.9%) with (95% CI: 40.79%, 49.1%).

### Factors associated with depression among PLWH

In bivariate regression analysis marital status, living condition, lost job, HIV related stigma, social support, CD4 count, side effect to HAART, WHO HIV/AIDS clinical stage, drug regimen and medication adherence were associated with depression at *p*-value =  < 0.25. These variables were candidate for further multivariable logistic regression model. In multivariable logistic regression analysis; BMI index, HIV/AIDS related stigma, side effect to HAART, level of income and WHO HIV/AIDS clinical stage II factors were associated with depression (Table 3).

**Table 3:** Factors associated with depression among People Living with HIV attending ART clinic in public Hospitals, Bale zone, Oromia, South East Ethiopia, 2021 (*n* = 554)

Variable	Depression status		COR(95%CI)	AOR(95%CI)
	**Depressed**	**Non depressed**		
**Age category**				
18–29	55	65	1.08(0.60, 1.94)	0.98(0.54, 1.78)
30–39	89	100	1.14(0.66, 1.96)	0.86(0.46,1.62)
40–49	73	99	0.94(0.54, 1.64)	0.66(0.29,1.46)
> = 50	32	41	1	1
**Marital status**				
Single	27	41	1	1
Married	165	180	1.39(0.820, 2.36)	1.59(0.78, 3.22)
Divorced	28	46	0.92(0.470, 1.81)	1.20(0.48, 2.99)
Widowed	29	38	1.15(0.584, 2.29)	1.24(0.51, 3.01)
**Level of education**				
unable to read and write	26	35	0.93(0.45, 1.91)	.57(0.20, 1.63)
able to read and write	52	53	1.23(0.65, 2.32)	.57(0.22, 1.47)
Primary school	86	81	1.33(0.74, 2.41)	.72(0.30, 1.75)
Secondary or preparatory	58	102	0.71(0.39, 1.30)	.35(0.15, 0.81)*
College or university	27	34	1	1
**Occupational status**				
Farmer	65	63	1	1
House wife	58	68	0.827(0.50, 1.35)	.87(0.46, 1.63)
Government employee	46	70	.637(0.383, 1.05)	.54(0.25, 1.17)
Daily laborers	54	61	.858(0.51, 1.42)	.87(0.45, 1.68)
Others	26	43	.586(0.32, 1.06)	.74(0.34, 1.60)
**Level of income**				
< 500	46	49	2.61(1.47, 4.61)*	3.1(1.59, 6.22)*
501–1500	61	76	2.23(1.31, 3.77)*	2.2(1.19, 4.04)*
1501–2500	72	48	4.17(2.42, 7.19)*	4.5(2.39, 8.59)*
2501–3500	38	43	2.45(1.35, 4.45)*	2.4(1.20, 4.85)/*
> 3501	32	89	1	1
**Body mass index**				
< 18.5	66	46	1	1
18.5–24.99	167	226	0.51(0.33, 0.78)*	0.49(0.29, 0.8) *
25- 29.99	16	33	0.33(0.16, 0.68)*	0.70(0.29, 1.69)
**Duration with ART**				
6-12 month	10	6	1	1
13–24 month	10	18	0.33(0.09, 1.19)	0.23(0.05, 1.08)
> 25 month	229	281	0.48(0.17, 1.36)	0.28(0.08, 1.04)
**HIV/AIDS related stigma**				
No	26	151	1	1
Yes	223	154	8.41(5.28, 13.37)*	8.2(4.96, 13.68)**
**Social support**				
Poor	177	204	1	1
Good	72	101	0.82(0.57, 1.18)	1.04(0.67, 1.62)
**Side effect to ART**				
Yes	68	86	1.24(1.71, 3.51)*	1.5(1.04, 2.51)*
No	181	219	1	1
**CD4 count**				
< 200	27	33	1.17(0.65, 2.12)	1.2(0.62, 2.49)
200–349	64	69	1.33(0.85, 2.09)	1.3(0.76, 2.29)
350–449	83	95	1.25(0.82, 1.90)	1.3(0.79, 2.20)
> 350	75	108	1	1
**WHO HIV clinical stage**				
Stage I	210	228	1	1
Stage II	21	47	0.48(0.28, 0.83)*	0.44(0.22, 0.9) *
Stage III	18	30	0.65(0.353, 1.20)	0.58(0.27, 1.25)
**Drug regimen**				
First line	201	246	1	1
Second line	48	59	1.0(0.65, 1.53)*	1.4(0.76, 2.47)
**HAART adherence**				
Poor	55	50	1.44(0.94, 2.21)	1.5(0.90, 2.61)
Good	194	255	1	1

*COR* Crude odd ratio, *AOR* Adjusted odds ratio, *CI* Confidence interval

^*^ *p*-value less than 0.05

^**^ *p*-value less than 0.001

## Discussion

This study provides important information about depression among HIV/AIDS patients in the settings studied. We found that 44.9% of study participants had depression. Participants who had different level of income, side effect to HAART, and having HIV related stigma were more likely suffer from depression. On the contrary, WHO clinical stage II, and those who had BMI normal cut of point (18.5–24.99) were 55.2% and 51% less likely to be depressed when compared to their counter parts, respectively.

The current study revealed that the prevalence of depression among HIV/AIDS patients was 44.9%. This is much higher when compared to the general population's depression rate of 17.5% [38]. The possible reason might be these groups are more likely to acquire depression as a result of the HIV related stigma. On the other hand, previously conducted studies in Ethiopia reported comparable prevalence of depression among people living with HIV such as Harar town east Ethiopia [24] and Tigray region [21] with the prevalence of 45.8% and 43.9% respectively. But Similar studies conducted in elsewhere reported in Cameron [39], Uganda [40] and Aksum town Tigray [22] were showed lower prevalence of 26.7%, 8.1%, 14.6% respectively. The possible difference could be attributed to different measurement tool, study population, sociocultural features participants, study setting and sample size differences.

However, the finding of this study was lower than the result obtained from studies conducted in Rwandan 81% [41], 63.1% in Khartoum, Sudan [42], 72.9% in Berger-Greenstein [43] and 79.1% in India [44] were found to be depressed. The reason for this discrepancy might be due to several factors, with difference of study setting, including the type of population being studied, the study periods, the depression screening instruments difference, and the sample size used.

In this study the odds of depression among respondents with perceived HIV related stigma was about 8 times higher compared to respondents who had no HIV related stigma. This finding was supported with studies conducted in Ethiopia at Alert Hospital, Addis Ababa [45] and at Hawassa University Comprehensive Specialized Hospital, Hawassa [46]. This might be having HIV related stigma associated with self-isolation which can increase a sense of depression symptoms and complicate relation with family, friend and other community members.

Depression was found to be substantially linked to those experiencing antiretroviral medication adverse effects. The odd of depression among respondents with side effect HAART was about 1.5 times higher compared to the counter parts. This finding is supported with studies reported in china [47, 48] this might indicates negative feelings regarding ART were exacerbated by the adverse effects, which led to an increase in depression symptoms.

Income level had statistically significant association with depressive symptoms as compared with those who had income level of ≥ 3501 ETB. Our finding consistent with studies done at North Showa, Ethiopia [49] and in Tigray, North Ethiopia [21] at Harar town eastern Ethiopia [24]. The possible explanation might be low income has been brought in patients in worries and stress to accomplish day to day activities this might contributes to develop depression symptoms.

In contrast to other studies conducted in India [44], at Hawassa University Comprehensive Specialized Hospital and Yirgalem Hospital, Ethiopia [26], Alert Hospital, Addis Ababa [45] and at Debrebirhan Referral Hospital, North Showa, Ethiopia [49]. WHO clinical HIV stage II 55.2% and those who had BMI normal cut of point (18.5–24.99) were 55.2% and 51% less likely to be depressed when compared to their counter parts, respectively.

### Limitation of the study

This study aimed to examine predictors of depression in a study area with no prior evidence on the distribution and associated risk factors. Although the study used primary data on level of depression, social support and perceived stigma with trained data collectors and supervisors, the findings must be interpreted in light of the following limitations. Firstly, due to the cross-sectional nature of the study, a cause-effect relationship cannot be established between the risk factors and depression. secondly, participants were assessed using self-reported surveys of depressive symptoms rather than gold-standard screening clinical interviews for the majority of depressive disorders. Furthermore, even though each self-reported measure of depressed symptoms has limits, there is evidence that the lack of anonymity in formal screening assessments may compromise accurate assessment of sensitive personal information such as depression symptoms thirdly, since it is hospital based, the finding may not be generalizable to the total population, Fourthly, data regarding social support and perceived stigma use were collected through selfreported questionnaires, which might be affected by the social desirability bias. Future studies could use more objective measurements and qualtative studies to further explore factors related to cultural aspects towards HIVAIDS.

## Conclusion

The prevalence of depression among people living with HIV in the study setting was high, almost two in every five HIV patients were depressed. Having perceived HIV related stigma side effect to HAART, and low-income level associated with depression among PLWH. Being having normal body mass index indicated less likely to have depressive symptoms. Health facilities should pay special attention, prioritize, assess and treat HIV patients who are experiencing depressed symptoms as their routine service. Counseling programs should be strengthened, and health education should be provided to patients and the community at large in order to reduce HIV-related stigma and poor social support. Further study with a qualitative study design and follow up studies would be recommended to support this finding.

## Data Availability

All data analysed during this study are included in this article.

## Abbreviations

AIDS: Acquired immune deficiency syndrome
ART: Antiretroviral therapy
HAART: Highly active antiretroviral treatments
HIV: Human immune virus
AOR: Adjusted odds ratio
PHQ-9: Patient health questions-9
SSQ-6: Social support questions-6
WHO: World health organization
CL: Confidence level
